# Pbx4 is Required for the Temporal Onset of Zebrafish Myocardial Differentiation

**DOI:** 10.3390/jdb3040093

**Published:** 2015

**Authors:** Robert M. Kao, Joel G. Rurik, Gist H. Farr, Xiu Rong Dong, Mark W. Majesky, Lisa Maves

**Affiliations:** 1 Center for Developmental Biology and Regenerative Medicine, Seattle Children’s Research Institute, 1900 9th Avenue, Seattle, WA 98101, USA; 2 Division of Cardiology, Department of Pediatrics, University of Washington, Seattle, WA 98105, USA

**Keywords:** cardiomyocyte, differentiation, myocardial morphogenesis, outflow tract, proepicardium, zebrafish, *pbx4*

## Abstract

Proper control of the temporal onset of cellular differentiation is critical for regulating cell lineage decisions and morphogenesis during development. Pbx homeodomain transcription factors have emerged as important regulators of cellular differentiation. We previously showed, by using antisense morpholino knockdown, that Pbx factors are needed for the timely activation of myocardial differentiation in zebrafish. In order to gain further insight into the roles of Pbx factors in heart development, we show here that zebrafish *pbx4* mutant embryos exhibit delayed onset of myocardial differentiation, such as delayed activation of *tnnt2a* expression in early cardiomyocytes in the anterior lateral plate mesoderm. We also observe delayed myocardial morphogenesis and dysmorphic patterning of the ventricle and atrium, consistent with our previous Pbx knock-down studies. In addition, we find that *pbx4* mutant larvae have aberrant outflow tracts and defective expression of the proepicardial marker *tbx18*. Finally, we present evidence for Pbx expression in cardiomyocyte precursors as well as heterogeneous Pbx expression among the pan-cytokeratin-expressing proepicardial cells near the developing ventricle. In summary, our data show that Pbx4 is required for the proper temporal activation of myocardial differentiation and establish a basis for studying additional roles of Pbx factors in heart development.

## 1. Introduction

Temporal control of cellular differentiation is essential for animal development. During cardiac development, timely activation of cardiomyocyte differentiation genes is needed for the proper morphogenetic formation of ventricular and atrial cardiac chambers [1,2]. In zebrafish cardiac development, cardiomyocyte differentiation is tightly linked with the morphogenetic movements needed for the bilateral cardiac primordia to undergo cell migration and fuse at the midline to form the heart tube [1,3–5]. Understanding the mechanisms behind cardiomyocyte differentiation are, thus, critical to understanding how the heart forms.

Recent studies have implicated Pbx homeodomain pioneer transcription factors and their Meis cofactor partners as critical regulators of heart development [6–11]. Pbx and Meis encode TALE (Three Amino acid Loop Extension)-class homeodomain-containing DNA-binding proteins. In mice and zebrafish, multiple *Pbx* genes are broadly expressed [12,13]. Well-characterized as cofactors for Hox proteins, Pbx/Meis also act as pioneer factors for Myod to promote skeletal muscle differentiation [14–16]. We previously showed, using antisense morpholinos (MOs) to knock down *pbx2* and *pbx4*, that *pbx2-MO;pbx4-MO* zebrafish embryos have delayed activation of cardiomyocyte differentiation [8]. The delayed differentiation and migration of myocardial precursor cells leads to defective myocardial morphogenesis and chamber formation in *pbx2-MO;pbx4-MO* embryos [8]. Our studies on heart and skeletal muscle support a model whereby Pbx proteins provide competence to respond to cell-lineage transcription factors to direct cellular differentiation programs.

In mice, *Pbx1/2/3* and *Meis1* are required for outflow tract development [6,7]. These studies showed that Pbx proteins act in neural crest cells to promote *Pax3* expression necessary for outflow tract development [7]. However, other studies have suggested roles for Pbx/Meis proteins within cardiomyocytes. We previously demonstrated that Pbx/Meis proteins can directly bind the promoter of the myocardial differentiation gene *myl7 in vitro* [8]. Postnatally in mice, *Meis1* is expressed in cardiomyocytes and promotes cardiomyocyte cell-cycle arrest [11]. PBX/MEIS binding sites are enriched in open chromatin in cardiac progenitor cell culture models, and Pbx binding sites are also associated with Tbx5 binding sites [9,10,17]. In spite of these studies, the requirements for Pbx factors in heart development have not yet been fully addressed.

Here, we present evidence that zebrafish *pbx4* mutant embryos display delayed onset of myocardial differentiation and morphogenesis, resembling the previously characterized zebrafish *pbx2/4* morpholino-knockdown phenotype [8]. Furthermore, we also demonstrate that *pbx4* is required for establishing a proper outflow tract and proepicardium, which gives rise to the epicardial mesothelium surrounding the heart. Finally, we provide evidence of Pbx expression in cardiomyocyte precursors and of heterogeneous Pbx expression in pan-cytokeratin-expressing proepicardial cells near the ventricle. Taken together, our results provide further evidence that Pbx proteins promote myocardial differentiation and suggest multiple roles for Pbx proteins in heart development.

## 2. Experimental Section

### 2.1. Zebrafish Husbandry

All experiments involving live zebrafish (*Danio rerio*) were carried out in compliance with Seattle Children’s Research Institute IACUC guidelines. Zebrafish were raised and staged as previously described [18]. Staging time refers to hours post fertilization (hpf) at 28.5 °C. In some cases, embryos were raised for periods at 24 °C. For studies prior to 24 hpf, somite (s) number was used for staging, and mutant and control embryos were somite-stage matched. In some cases, embryos were incubated in 1-phenyl-2-thiourea to inhibit pigmentation [18]. The wild-type stock and genetic background used was AB. The *pbx4^b557^* mutant strain was previously described and is likely a null allele [19]. *pbx4^b557^* genotyping was performed using forward primer 5′ACTCGGCGGACTCTCGCAAGC3′ and reverse primer 5′GGCTCTCGTCGGTGATGGCCATGATCT3′. The genotyping PCR product is 128 base pairs, and digesting with XbaI yields a 98 base pair product from the mutant allele. The *Tg(myh6:EGFP)^s958^, Tg(myl7:EGFP)^twu34^*, and *Tg(myl7:h2afva-mCherry)^sd12^* strains have been described [20–22].

### 2.2. Whole-mount RNA in Situ Hybridization

The following cDNA probes were used: *krox20* (*egr2b*-Zebrafish Information Network) [23]; *tnnt2a* [24]; *myl7* [25]; *vmhc* [25]; *myh6* [26]; *nppa* [26]; *gata4* [27]; *gata5* [27]; *hand2* [28]; *elnb* [29]; *tbx18* (MGC:194980); and *ltbp3* [30]. Whole-mount *in situ* hybridization colorimetric and fluorescent *in situ* staining was performed as previously described [15,31], with the following modifications. For colorimetric NBT/BCIP stained embryos, dimethylformamide was used prior to stepwise glycerol clearing to 80% glycerol in 1× PBS. Antisense RNA probes for either colorimetric or fluorescent *in situ* hybridization experiments were diluted into 5% dextran sulfate hybridization buffer. To reduce non-specific colorimetric staining at 48 hpf and later stages, we immediately processed 4% PFA/1× PBS-fixed embryos for de-pigmentation using 1 part 0.1% KOH (vol.): 1 part 1× PBS-0.1% Tween (vol.): 0.1 part 30% hydrogen peroxide (vol.) for 3–4 h at room temperature with gentle agitation. After 1 µg/mL proteinase K digestion and post-fixation steps, 48 hpf and later stages were then stored in pre-hybridization buffer at −20 °C overnight until further use. Following staining and imaging, tail clips from post-*in situ* hybridized embryos were lysed and genotyped for *pbx4* as above.

### 2.3. Whole-mount Zebrafish Immunostaining and Cardiomyocyte Cell Counting

Whole-embryo immunostaining was performed with the following primary antibodies: anti-Pbx (1:100, rabbit antisera, [32]), MF20 (1:50, supernatant, Developmental Studies Hybridoma Bank, University of Iowa), anti-GFP (1:500, Roche #11814460001), anti-mCherry (1:500, Rockland Antibodies & Assays #600-401-P16), anti-GFP (1:500, Abcam ab13970), and pan-cytokeratin (1:100, Sigma C2562). Secondary antibodies were goat anti-rabbit 568 (1:800, Life Technologies #A-11011), goat anti-rabbit 594 (1:500, Life Technologies #R37117), goat anti-chicken 488 (1:500, Life Technologies #A-11039), and goat anti-mouse 488 (1:800, Life Technologies #A-11029). Whole-mount immunostaining was performed as previously described [33]. For anti-Pbx staining at 48 hpf and for anti-pan-cytokeratin staining, Dent’s fixative (cold 80% methanol and 20% DMSO; [34]) was used with overnight fixation at 4 °C, and a modified whole-mount staining protocol was used [35].

Cardiomyocyte nuclei were counted using *Tg(myl7:h2afva-mCherry)^sd12^* [21] in *pbx4^b557^−/−* and control sibling embryos. 26 hpf embryos were immunostained and whole mounted in 100% glycerol. 48 hpf embryo hearts were removed after immunostaining via microdissection and mounted in 30% glycerol/70% PBS-0.1% Tween. Nuclei were counted using ImageJ software [36]. Cell number data were analyzed using a Student’s *t*-test (2-tailed, equal variance).

### 2.4. Fluorescent Confocal Microscopy and Stereoscope Imaging

Fluorescent confocal microscopy (Leica SP5) was used for examining embryos using a 20× air objective (NA = 0.7). In instances where longer working distances were necessary, fluorescently stained specimens were imaged with an upright Olympus fluorescent single photon confocal equipped with a 20× water-dipping objective (NA = 0.95). For 12 s and 16 s stage fluorescent RNA *in situ*, ImageJ was used for pairwise stitching of original optical stacks taken with 20× air objective (NA = 0.7), using the method previously described [37].

## 3. Results

### 3.1. Zebrafish Pbx4 Mutants Exhibit Defective Heart Function

**Figure 1:**
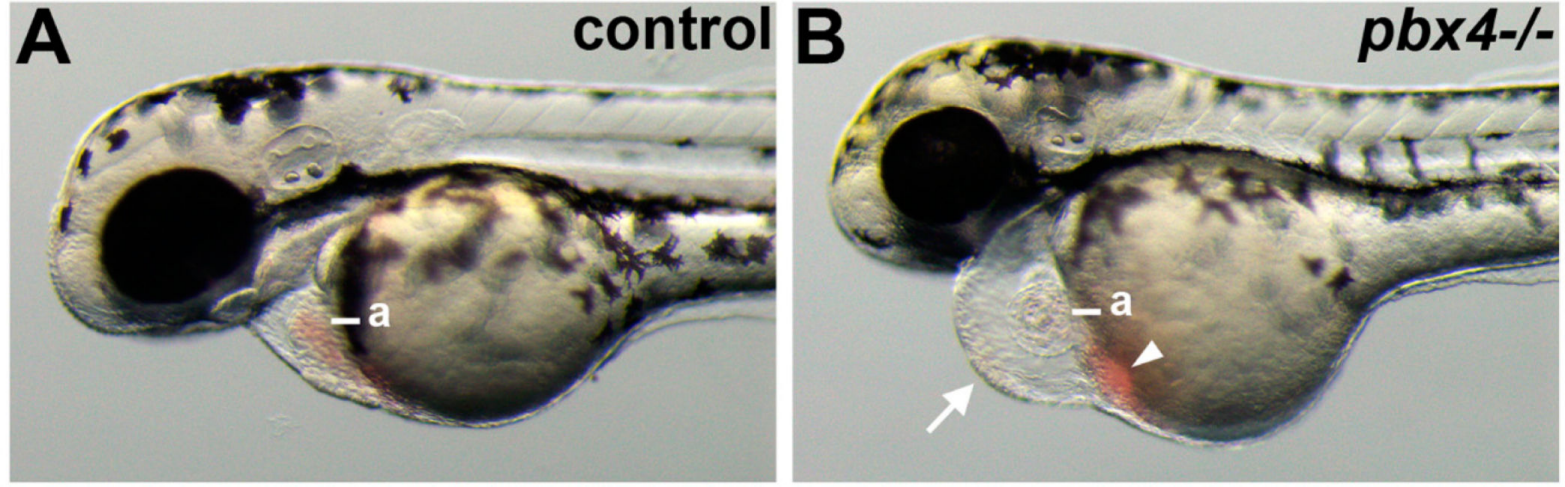
Defective heart development in *pbx4* mutant embryos. (**A,B**) Lateral views of live (**A**) control and (**B**) *pbx4^b557^−/−* embryos at 50 h post fertilization (hpf). *pbx4^b557^* mutant embryos show pericardial edema (arrow in **B**) and blood pooled near the atrium (arrowhead in **B**). a, atrium. For controls, *n* = 23. *pbx4^b557^* mutant embryos (*n* = 20) all show similar phenotypes as in (**B**). Anterior is to the left.

Pöpperl *et al.* [19] previously described *pbx4^b557^* mutant embryos as having a swollen pericardium and thin, weakly beating heart, and we showed that *pbx2-MO;pbx4-MO* embryos also have pericardial edema and decreased heart rate [8]. We confirmed that *pbx4^b557^* mutant embryos have pericardial edema and exhibit blood pooled by the atrium (Figure 1B). We assessed heart rates and found that while control 50 hpf sibling embryos had 135 ± 11 beats/min (*n* = 14), *pbx4^b557^* mutant embryos had 105 ± 14 beats/min (*n* = 13). Thus, zebrafish Pbx4 is needed for proper heart development and function, consistent with the previous studies [8,19].

### 3.2. Pbx4 is Required for Proper Temporal Onset of Cardiac Muscle Differentiation

**Figure 2:**
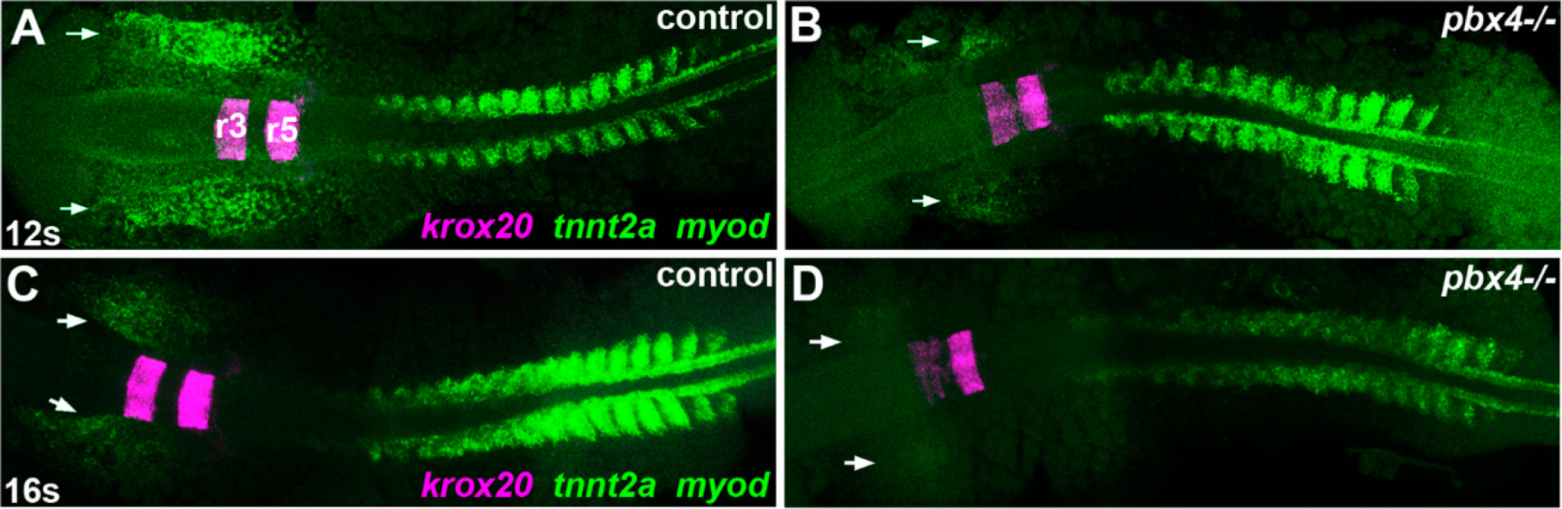

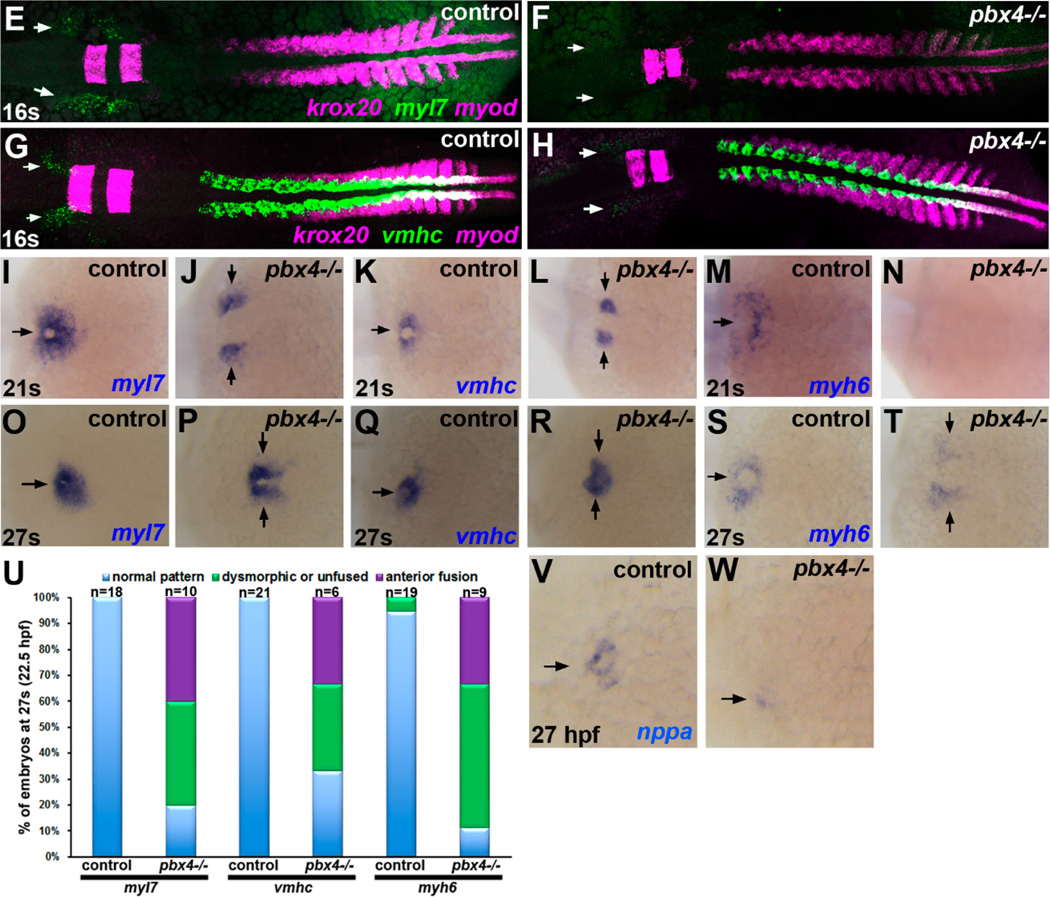
Delayed onset of cardiomyocyte differentiation genes in *pbx4* mutant embryos. (**A–T, V–W**) RNA *in situ* expression of cardiomyocyte differentiation genes (**A–D**) *tnnt2a*, (**E–F, I–J, O–P**) *myl7*, (**G–H, K–L, Q–R**) *vmhc*, (**M–N, S–T**) *myh6*, and (**V–W**) *nppa* in (**A,C,E,G,I,K,M,O,Q,S,V**) control and (**B,D,F,H,J,L,N,P,R,T,W**) *pbx4^b557^−/−* embryos. Developmental stages are indicated. Embryos are shown in dorsal view, anterior towards the left. (**A–H**) Expression of the Pbx-dependent gene *krox20*, marking rhombomeres 3 and 5 (r3, r5 in **A**) in the hindbrain, was included to distinguish between control and *pbx4^b557^−/−* mutants [12,19]. Expression of *myod*, which is expressed in a stripe in each somite plus two additional pre-somitic stripes [38], was included for somite staging. Arrows indicate ALPM expression domains of cardiomyocyte differentiation genes. (**A,B**) At 12 s, *tnnt2a* is absent or reduced in *pbx4^b557^−/−* embryos (*n* = 8; 6/8 absent, 2/8 reduced) compared to controls (*n* = 30; all similar). (**C,D**) at 16 s, *tnnt2a* is reduced in *pbx4^b557^−/−* embryos (*n* = 7) compared to controls (*n* = 28). (**E,F**) at 16 s, *myl7* is absent or reduced in *pbx4^b557^−/−* embryos (*n* = 9; 3/9 absent, 6/9 reduced) compared to controls (*n* = 22). (**G,H**) at 16 s, *vmhc* is absent or reduced in *pbx4^b557^−/−* embryos (*n* = 6; 1/6 absent, 5/6 reduced) compared to controls (*n* = 10). (**I–T**) At 21 s and 27 s, *myl7, vmhc*, and *myh6* show delayed fusion and abnormal patterning of expression domains in *pbx4^b557^−/−* embryos compared to controls. For (**I–N**), numbers of affected embryos are provided in the text. For (**O–T**), numbers of affected embryos are graphed in (**U**). Arrows indicate expression domains of cardiomyocyte differentiation genes. (**U**) Graph displaying percentage of embryos at 27 s with either normally patterned (blue), dysmorphic or unfused (green), or anteriorly fused (purple) cardiac primordia. (**V–W**) At 27 hpf, *nppa* is reduced in *pbx4^b557^−/−* embryos (*n* = 4, all similar reduced expression) compared to controls (*n* = 24). Arrows indicate myocardial expression domains.

To determine whether Pbx4 is required for the initiation of myocardial differentiation, we examined the onset of expression of early myocardial differentiation genes in *pbx4^b557^* mutant embryos. At the 12 somite (12 s) stage (15 hpf), *cardiac troponin T type 2a* (*tnnt2a*) is absent or very reduced in cardiomyocyte precursors in the anterior lateral plate mesoderm (ALPM) of *pbx4^b557^−/−* embryos compared to control embryos (Figure 2A,B). By 16 s (17 hpf), *tnnt2a* expression is still reduced in *pbx4^b557^−/−* embryos compared to controls (Figure 2C,D). Two other early myocardial differentiation genes, pan-myocardial *myl7*, and ventricular *vmhc* [25], are also absent or very reduced in the ALPM of *pbx4^b557^−/−* embryos at 16 s (Figure 2E–H). At the time of fusion of bilateral cardiomyocyte populations at 21 s (19.5 hpf), the majority of *pbx4^b557^−/−* embryos display delayed fusion of *myl7*-expressing cardiac primordia (6/7 embryos not fused, Figure 2J), compared to controls (1/13 embryos not fused, Figure 2I). Similarly, *pbx4^b557^−/−* embryos display non-fused domains of *vmhc*-expressing cells (6/7 embryos not fused, Figure 2L) compared to controls (2/18 not fused, Figure 2K). Finally, analysis of atrial *myh6*-expressing cardiomyocyte precursors revealed *pbx4^b557^−/−* embryos with non-fused and diminished expression (4/5 embryos not fused, Figure 2N) compared to controls (3/17 not fused, Figure 2M). To determine whether the delayed cardiac primordia midline fusion phenotype at 21 s was due to a myocardial morphogenesis defect and not a general developmental delay of *pbx4^b557^−/−* embryos, we assayed these cardiomyocyte markers at 27 s (22.5 hpf). We find that *pbx4^b557^−/−* embryos continue to display dysmorphic patterning of *myl7, vmhc*, and *myh6* expression domains compared to controls (Figure 2O–T). In particular, myocardial domains in 27 s *pbx4^b557^−/−* embryos can remain not fused or show anterior rather than normal posterior fusion ([4]; Figure 2O–U). These data are consistent with a delayed initiation of myocardial differentiation and subsequent morphogenesis defect of cardiomyocyte primordia in *pbx4^b557^−/−* embryos.

To further investigate the myocardial differentiation defects in *pbx4^b557^−/−* embryos, we examined expression of *nppa*, a myocardial differentiation gene whose expression is absent in *pbx2-MO;pbx4-MO* embryos [8]. *nppa* initially turns on at about 24 hpf in control embryos [8]. At 27 hpf, *nppa* expression is reduced in *pbx4^b557^−/−* embryos (Figure 2V,W), although it does not appear as strongly reduced as was observed in *pbx2-MO;pbx4-MO* embryos [8]. Taken together, these results show that the myocardial differentiation phenotype in *pbx4^b557^−/−* embryos is similar to, although possibly not as severe as, the myocardial differentiation phenotype we previously characterized in *pbx2-MO;pbx4-MO* embryos [8].

### 3.3. Early Myocardial Specification is Not Reduced in Pbx4^b557^−/− Embryos

**Figure 3:**
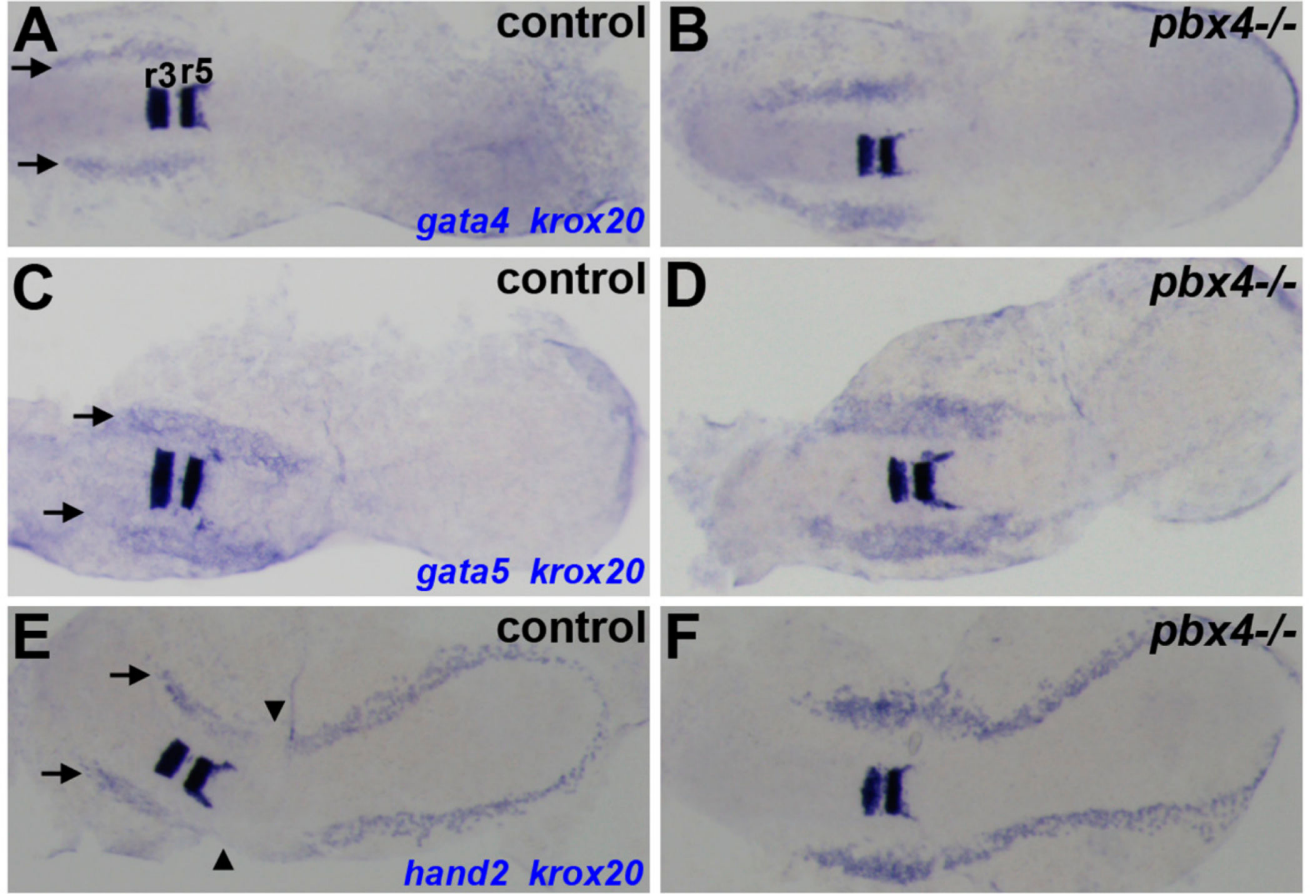
Early cardiomyocyte specification does not appear reduced in *pbx4* mutant embryos. (**A–F**) RNA *in situ* expression at the 10 s stage of cardiomyocyte specification genes (**A–B**) *gata4*, (**C–D**) *gata5*, and (**E–F**) *hand2* in the anterior lateral plate mesoderm (ALPM) in (**A,C,E**) control and (**B,D,F**) *pbx4^b557^−/−* embryos. Expression of the Pbx-dependent gene *krox20*, marking hindbrain rhombomeres 3 and 5 (r3, r5 in **A**), was included to distinguish between control and *pbx4^b557^−/−* embryos [12,19]. Arrows indicate ALPM expression domains in control embryos. The junction between *hand2*-expressing ALPM and posterior lateral plate mesoderm domains is noted by arrowheads (**E**). For (**A–D**), *n* ≥ 10 for each marker in *pbx4^b557^−/−* embryos and *n* ≥ 30 for each marker in control embryos, all with similar expression patterns. For (**E–F**), *pbx4^b557^−/−* embryos display expanded *hand2* expression (*n* = 14; 13/14 with expanded expression) compared to control embryos (*n* = 47; all similar). Embryos are shown in dorsal view, anterior towards the left.

We previously showed in *pbx2-MO;pbx4-MO* embryos that, while myocardial differentiation is reduced, myocardial specification appears normal [8]. We, therefore, investigated early myocardial specification in *pbx4^b557^−/−* embryos. We find that myocardial specification genes *gata4, gata5*, and *hand2* are robustly expressed in *pbx4^b557^−/−* embryos at 10 s (Figure 3). In particular, we observe an expanded domain of *hand2* expression, similar to what we previously reported in *pbx2-MO;pbx4-MO* and *hand2-MO* embryos (Figure 3E,F) [8]. These results show that, in contrast to myocardial differentiation markers, myocardial specification markers do not show reduced expression in *pbx4^b557^−/−* embryos.

### 3.4. Pbx4 is Required for Cardiac Chamber Morphogenesis, Proper Outflow Tract Development, and Proepicardial Development

**Figure 4:**
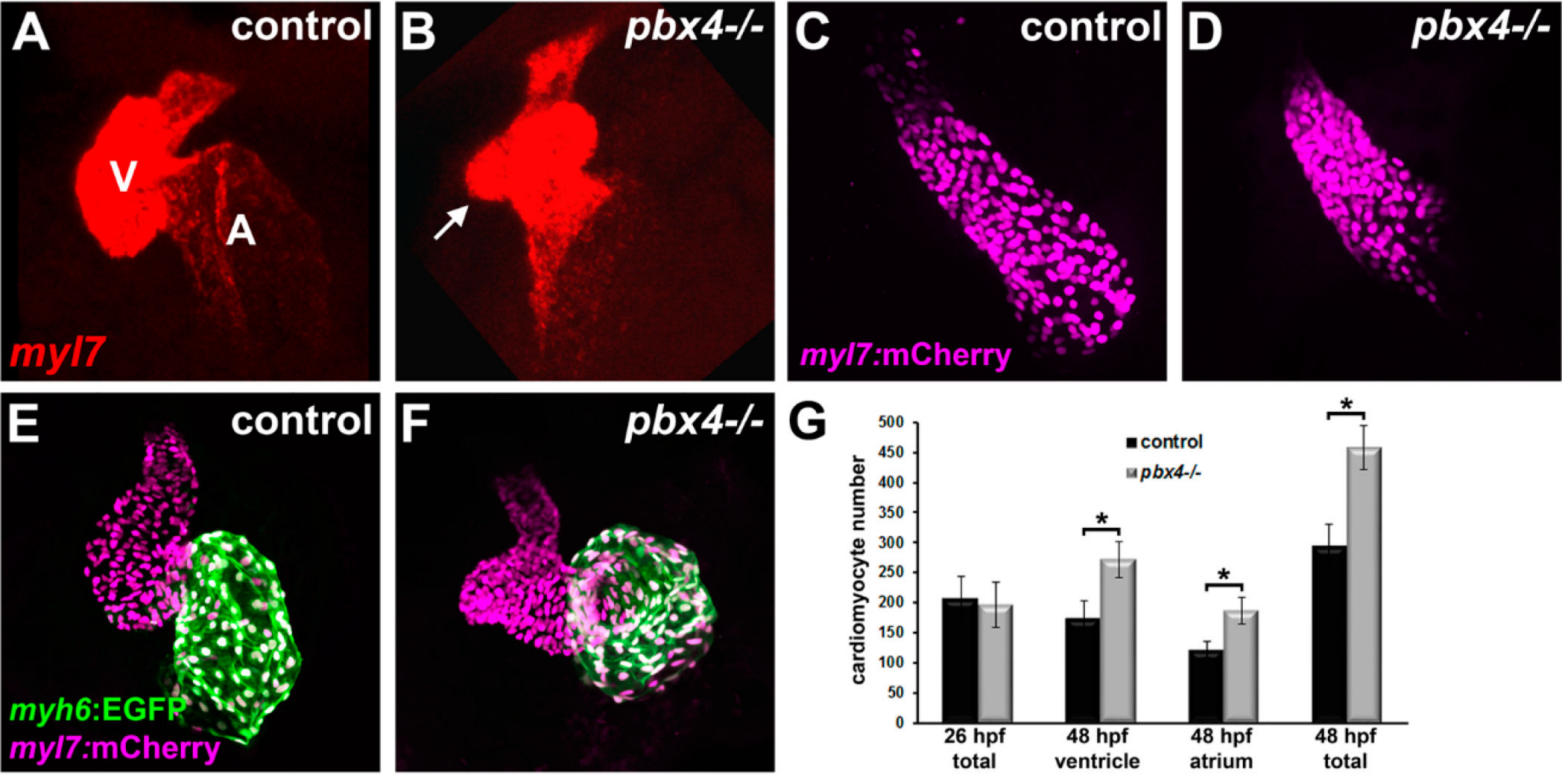

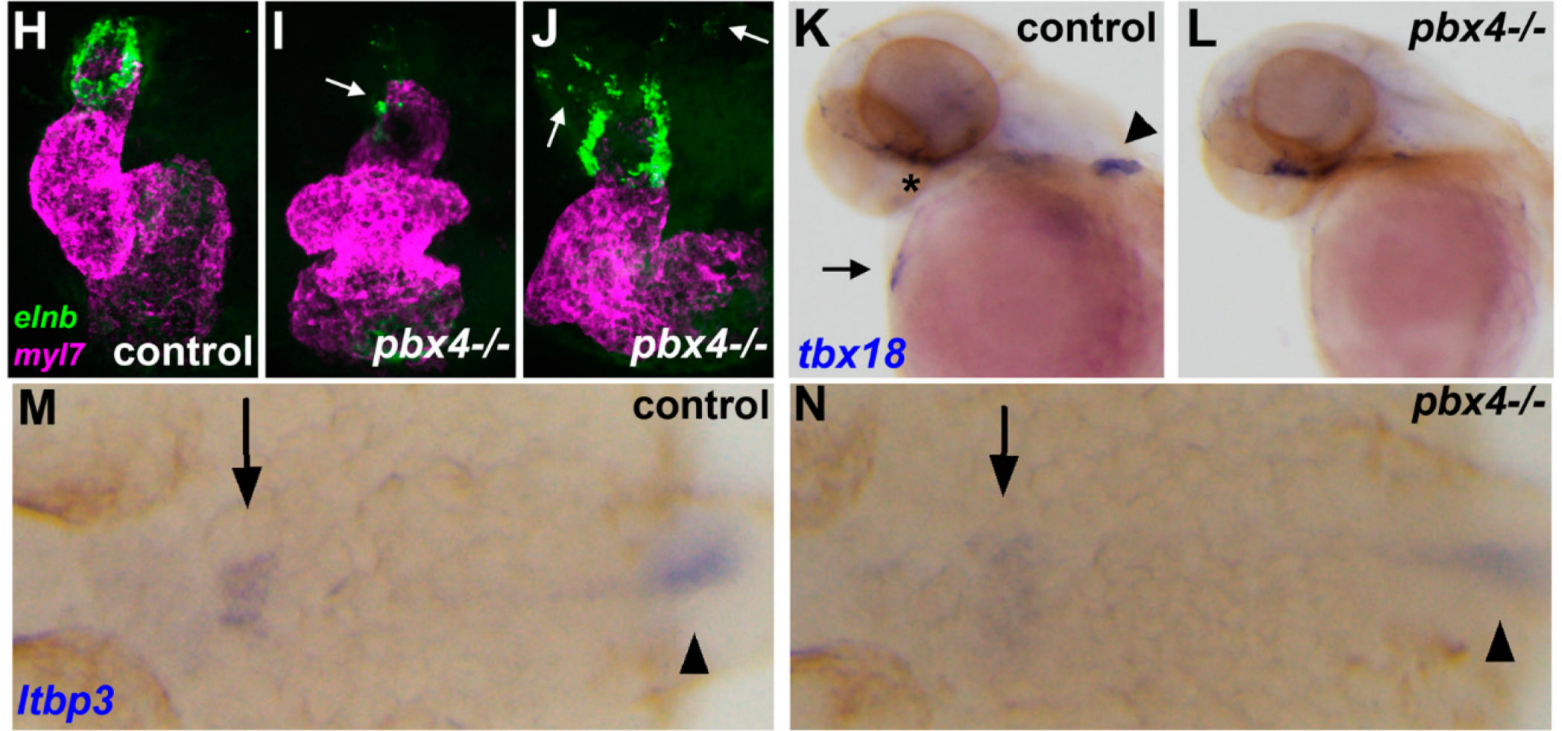
Cardiac chamber, outflow tract, and proepicardial development defects in *pbx4* mutant embryos. (**A,B**) RNA *in situ* expression of cardiomyocyte differentiation gene *myl7* (red) at 48 hpf in (**A**) control (*n* = 16) and (**B**) *pbx4^b557^−/−* (*n* = 4) embryos. V, ventricle. A, atrium. Arrow in (**B**) points to abnormal bulge in *pbx4^b557^−/−* ventricle. (**C,D**) Cardiomyocyte nuclei (magenta) in 26 hpf *Tg(myl7:h2afva-mCherry)^sd12^* (**C**) control (*n* = 13) and (**D**) *pbx4^b557^−/−* (*n* = 9) embryos. (**E,F**) Cardiomyocyte nuclei (magenta) in 48 hpf *Tg(myl7:h2afva-mCherry)^sd12^* (**E**) control (*n* = 9) and (**F**) *pbx4^b557^−/−* (*n* = 7) embryos. *Tg(myh6:EGFP)^s958^* (green) is used to identify atrial cells. (**G**) Graph displaying cardiomyocyte nuclei count data at 26 hpf and 48 hpf. Error bars represent standard deviation. * *P* < 0.00003. (**H,J**) RNA *in situ* expression of outflow tract smooth muscle marker *elnb* (green) and *myl7* (magenta) at 72 hpf in (**H**) control and (**I,J**) *pbx4^b557^−/−* embryos. In control embryos (*n* = 15), *elnb* expression appears as a ring (**H**). In *pbx4^b557^−/−* embryos, *elnb* expression can appear reduced (arrow in **I**; 5/9 embryos) or expanded and bifurcated (arrows in **J**; 4/9 embryos). (**K,L**) RNA *in situ* expression of *tbx18* at 48 hpf in (**K**) control and (**L**) *pbx4^b557^−/−* embryos. *tbx18* expression in pectoral fin (arrowhead) and proepicardial cells (arrow) is lost in *pbx4^b557^−/−* embryos (*n* = 9) compared to controls (*n* = 11), while facial expression (asterisk) is maintained. Hearts and embryos are shown in ventral view, anterior toward the top. (**M,N**) RNA *in situ* expression of *ltbp3* at 24 hpf in (**M**) control and (**N**) *pbx4^b557^−/−* embryos. *ltbp3* expression in second heart field domain (arrows) appears broader yet weaker in *pbx4^b557^−/−* embryos (*n* = 10, all weaker expression) compared to controls (*n* = 45, all similar), while notochord expression (arrowheads) is maintained. Embryos are shown in dorsal view, anterior towards the left.

Our previous analyses showed that *pbx2-MO;pbx4-MO* embryos have dysmorphic ventricular and atrial chambers [8]. We examined *pbx4^b557^−/−* embryos and found that they also had dysmorphic chambers (Figure 4A,B). In particular, we observe variable bulges in the ventricle (arrow in Figure 4B; also see Figure 4F,I,J). These dysmorphic chambers are similar to what we observed in *pbx2-MO;pbx4-MO* embryos and may be due, at least in part, to the delayed and abnormal heart tube morphogenesis (Figure 2) [8]. To assess the basis of this chamber dysmorphogenesis, we counted cardiomyocytes at two stages, 26 hpf and 48 hpf, using the transgenic strain *Tg(myl7:h2afva-mCherry)^sd12^*, which labels all myocardial nuclei [21]. At 26 hpf, we find no significant difference between *pbx4^b557^−/−* embryos and control siblings (Figure 4C,D,G). At 48 hpf, we find that both ventricular and atrial chambers show increased numbers of cardiomyocytes in *pbx4^b557^−/−* embryos compared to controls (Figure 4E–G). We also find that patterning of the outflow tract is disrupted in *pbx4^b557^−/−* embryos (Figure 4H–J). The outflow tract defects show variability: the outflow tract smooth muscle marker *elastinb* (*elnb*; [29]) is reduced in some *pbx4^b557^−/−* embryos (Figure 4I) and appears expanded in some *pbx4^b557^−/−* embryos (Figure 4J). Furthermore, we find evidence that Pbx4 is needed for proepicardial development, because *pbx4^b557^−/−* embryos show loss of expression of *tbx18*, a key marker of the proepicardium (Figure 4K,L) [39,40]. While our findings do not directly reveal the cause of the chamber and outflow tract dysmorphogeneis, these results suggest that Pbx4 may have multiple roles in heart development: an early role in promoting myocardial differentiation and morphogenesis, and later roles in repressing myocardial differentiation and regulating outflow tract and proepicardial formation.

Defects in zebrafish outflow tract development and *elnb* expression have been linked with defects in the second heart field (SHF), a source of later-differentiating myocardial progenitors after the initial cardiac tube is formed [30,41–45]. In particular, loss of the SHF marker *ltbp3* leads to a reduced outflow tract and reduced *elnb* expression, while increased *ltbp3* expression, seen in zebrafish embryos lacking *cadm4*, correlates with an expanded outflow tract [30,44,46]. We find that expression of *ltbp3* is disrupted in *pbx4^b557^−/−* embryos (Figure 4M,N). However, in contrast to the varied patterns of *elnb* expression in *pbx4^b557^−/−* embryos (Figure 4H–J), we see a consistent, diffuse, defective *ltbp3* expression pattern that appears broader but also weaker than that in controls (Figure 4M,N). Thus, these findings suggest that, while there appear to be defects in the formation of the SHF in *pbx4^b557^−/−* embryos, it is not yet clear how these defects lead to the variable outflow tract defects in *pbx4^b557^−/−* embryos.

### 3.5. Pbx Expression Domains Support Multiple Roles for Pbx Proteins in Heart Development

**Figure 5:**
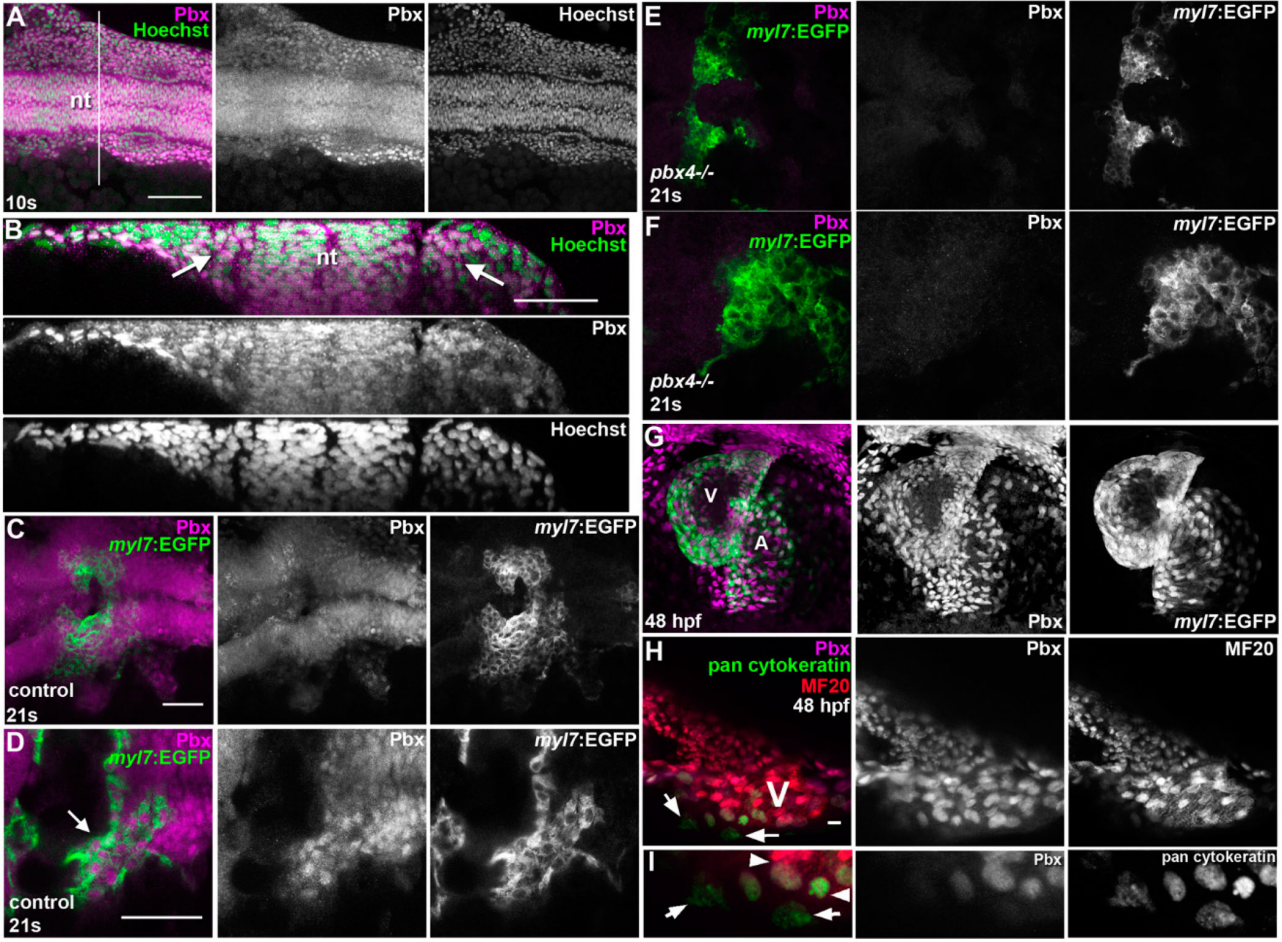
Analysis of Pbx expression during zebrafish cardiac development. (**A–G**) Pbx immunostaining at (**A–B**) 10 s (14 hpf), (**C–F**) 21 s (19.5 hpf), and (**G–H**) 48 hpf. (**A–B**) Pbx expression (magenta) in wild type 10 s embryo co-stained with Hoechst to label nuclei (green). (**A**) Dorsal view, anterior to the left. The line in (**A**), approximately at the level of hindbrain rhombomere 3, shows where we took an optical cross-section view for (**B**). Arrows in (**B**) show Pbx expression in ALPM cells next to the neural tube (nt in **A,B**). (**C–F**) Pbx expression (magenta) in *Tg(myl7:EGFP)^twu34^* (green) 21 s (**C–D**) control embryo and (**E–F**) *pbx4−/−* embryo, at low (**C,E**) and high (**D,F**) magnification. Arrow in (**D**) points to cells co-expressing *myl7*:EGFP and Pbx. Dorsal view, anterior to the left. (**G**) Pbx expression (magenta) in *Tg(myl7:EGFP)^twu34^* (green) 48 hpf embryo. Ventral view, anterior to the top. V, ventricle. A, atrium. (**H–I**) Pbx expression (magenta) in wild-type 48 hpf embryo co-stained for proepicardial pan-cytokeratin expression (green) and myocardial cells (MF20, red), at low (**H**) and high (**I**) magnification. V, ventricle. Arrows in (**H,I**) point to two pan-cytokeratin-expressing cells that do not express Pbx, compared to neighboring cardiomyocytes and pan-cytokeratin/Pbx co-expressing cells (arrowheads, **I**). Scale bar for (**A**): 100 µm. Scale bar for (**B–D**): 50 µm. Scale bar for (**H**): 10 µm.

The expression of Pbx proteins in premigratory neural crest cells has been shown to contribute to the outflow tract defects observed in mouse *Pbx1* mutants, and Pbx proteins are also expressed in myocardial cells in the mouse embryo outflow tract, but Pbx expression in cardiomyocyte precursors has not been fully addressed [6,7]. To examine Pbx expression, we used a Pbx antiserum that was raised against zebrafish Pbx4 and crossreacts with Pbx2 [32]. At the 10 s stage, wild-type zebrafish embryos show nuclear Pbx expression in the ALPM and largely throughout the embryo (Figure 5A,B). At later stages, we used the *Tg(myl7:EGFP)^twu34^* reporter to identify differentiated cardiomyocytes with nuclear Pbx expression at 21 s (19.5 hpf; Figure 5C,D) and 48 hpf (Figure 5G). While overall the Pbx staining appears predominantly nuclear, there may be some cytoplasmic Pbx expression as well (Figure 5D, for example). We find that, at 21 s, the Pbx immunostaining signal is largely absent from *pbx4−/−* embryos (Figure 5E,F), confirming that the Pbx antibody is predominantly recognizing Pbx4. These findings support a role for Pbx proteins acting in early cardiomyocytes to promote their differentiation. We also find heterogeneous expression of Pbx in the pan-cytokeratin-expressing proepicardial cells at 48 hpf near the ventricle of the heart (Figure 5H,I) [47]. Taken together, these results provide evidence of Pbx expression in early cardiomyocyte precursors, in differentiated cardiomyocytes, and in spatially-restricted pan-cytokeratin-expressing regions, supporting multiple roles for Pbx proteins in heart development.

## 4. Discussion

### 4.1. Pbx Proteins Promote Early Myocardial Differentiation and Morphogenesis

Our study provides support for an early role for Pbx proteins in promoting myocardial differentiation by showing that *pbx4* mutant embryos have delayed activation of myocardial differentiation (Figure 2) and that Pbx proteins are expressed in both cardiomyocyte precursors and differentiated cardiomyocytes (Figure 5). The delayed onset of cardiomyocyte differentiation markers and the delayed morphogenesis of myocardial cells during cardiac fusion and heart tube formation that we observe in *pbx4* mutant embryos also provides further support that myocardial differentiation and morphogenesis are intimately linked [1,4,48,49]. Studies in mice and other model organisms have revealed varied roles for Pbx proteins in promoting and inhibiting cellular differentiation in many developmental contexts [13,50]. Studies of Pbx functions in skeletal muscle have demonstrated the critical role of Pbx proteins in promoting skeletal muscle differentiation, in particular through acting as pioneer factors for Myod [14–16,51–54]. Our studies here not only demonstrate that Pbx proteins promote early myocardial differentiation, but also reveal that *pbx4* appears to inhibit later myocardial differentiation. Even though we have shown that Pbx proteins can bind the zebrafish *myl7* promoter [8], how Pbx4 activates or represses myocardial differentiation genes at different stages of development is not yet known.

It is not yet clear whether the role of Pbx proteins in promoting early myocardial differentiation is conserved. Previous studies in mouse embryos, using combinations of *Pbx* gene null alleles, have shown that *Pbx* genes are required for outflow tract development [6,7], and we show that zebrafish *pbx4* is needed for outflow tract development (Figure 4). However, because of early lethality of certain allelic combinations, the full requirements for Pbx proteins in mouse heart development have not yet been determined. Thus, studies have not yet directly addressed whether early myocardial differentiation is affected in Pbx-deficient mice.

Pbx proteins have overlapping expression patterns and redundant roles [6,12,55–57]. In our previous studies, we used antisense morpholinos (MOs) to knock down both zebrafish *pbx2* and *pbx4*, and we showed that *pbx2-MO;pbx4-MO* zebrafish embryos have delayed differentiation and migration of myocardial precursor cells and defective myocardial morphogenesis and chamber formation [8]. Our current study demonstrates that *pbx4^b557^−/−* embryos also have delayed cardiomyocyte differentiation and morphogenesis. The myocardial differentiation phenotype appears less severe in *pbx4^b557^−/−* embryos than in *pbx2-MO;pbx4-MO* embryos, pointing to a requirement for *pbx2* in addition to *pbx4* in myocardial differentiation. For example, *nppa* expression, which is absent in *pbx2-MO;pbx4-MO* embryos at 28 hpf [8], is present but reduced in *pbx4^b557^−/−* embryos (Figure 2). It is possible that the variability that we observe in *pbx4^b557^−/−* embryos, for example in the outflow tract phenotype (Figure 4), is due to variable compensation by other *pbx* genes. Therefore, in order to fully address the functions of Pbx proteins in heart development, it will be critical to examine zebrafish mutant combinations for additional *pbx* genes in future studies. Indeed, our preliminary studies show that zebrafish *pbx2;pbx4* double mutant embryos exhibit more severe and more consistent defects in myocardial differentiation and outflow tract formation than *pbx4* mutants [58]. Recent studies have found that zebrafish morpholino knockdown and mutant phenotypes often do not correspond [59,60]. While our zebrafish *pbx* gene morpholino and mutant studies do generally correspond, to fully address *pbx* gene requirements, we favor the use of mutant strains for future zebrafish *pbx* gene studies.

### 4.2. Pbx4-dependent Patterning of the Developing Outflow Tract

Previous studies have shown that loss of different combinations of *Pbx* genes in mouse embryos can lead to a spectrum of outflow tract defects [6,7]. These studies in mouse embryos also demonstrated that Pbx proteins are expressed in many cell types involved in outflow tract development. In particular, Pbx1 expression in pre-migratory cardiac neural crest cells directly activates expression of *Pax3*, which is required for cardiac neural crest cells to properly contribute to outflow tract development [7,61]. Pbx proteins are also expressed in vascular smooth muscle cells of the outflow tract and great arteries in mouse embryos [6,7]. We find that zebrafish *pbx4* is required for proper patterning of the smooth muscle *elnb*-expressing domain of the outflow tract (Figure 4). In contrast to the loss of *elnb* expression in the outflow tract observed in loss-of-function studies for zebrafish *ltbp3, nkx2.5*, and *tbx1* [30,44,45], we observed a variable mispatterning of the *elnb* domain along the outflow tract in *pbx4* mutants (Figure 4). Loss of zebrafish *cadm4* function leads to an expansion of the outflow tract, although *elnb* expression was not examined [46]. In future studies, it will be important to determine whether *pbx4* is acting upstream or downstream of *tbx1* and these other factors involved in outflow tract development.

Our results point to two potential mechanisms by which *pbx4* might regulate outflow tract development. First, we observe an expansion of *hand2* expression in the ALPM of *pbx4* mutants (Figure 3). Early SHF cells arise from *hand2*-expressing cells in the early ALPM [41,44]. The expanded *hand2* expression domain in *pbx4* mutants could, thus, provide a possible source of additional SHF cells and, indeed, our results with *ltbp3* expression indicate a possible expanded SHF (Figure 4). An expanded SHF could lead to the increased cardiomyocytes that we observe at 48 hpf as well as the expanded *elnb* expression that we observe in some *pbx4* mutant embryos (Figure 4). Second, the opposite effects that we observe in *pbx4* mutants on early *versus* later cardiomyocyte differentiation is similar to the dual effects of bone morphogenetic protein (BMP) signaling on early *versus* later cardiomyocyte differentiation, and BMP signaling regulates outflow tract formation [41,62,63]. In future studies, it will be important to examine how *pbx4* regulates *hand2* expression and interacts with BMP signaling.

A recent study has found that Pbx1 directly regulates PDGFRβ expression in vascular smooth muscle progenitors in mouse kidney development [64]. PDGFRβ expression is also required for coronary vascular smooth muscle development and for cardiac neural crest cell contributions to outflow tract development in mouse embryos [65–67]. Whether PDGFRβ regulation is conserved in zebrafish and contributes to the phenotypes we observe in *pbx4* mutants remains to be determined. Genetic mosaic studies in zebrafish embryos should help establish in which cell types Pbx4 functions to regulate outflow tract development. Studies implicating Pbx proteins in outflow tract malformations in human congenital heart defects further underscore the importance of understanding how Pbx proteins function in heart development [68].

### 4.3. Pbx Expression Heterogeneity among Pan-Cytokeratin-Expressing Cells Adjacent to the Ventricle

Previous studies have demonstrated broad expression of Pbx proteins in many cell types involved in heart development [6,7]. Our zebrafish Pbx immunostaining data is in agreement with these studies and other previous reports of broad Pbx expression (Figure 5) [13,19]. While overall our Pbx immunostaining appears predominantly nuclear, there may also be some cytoplasmic Pbx expression (Figure 5). Nuclear and cytoplasmic expression of Pbx would be consistent with earlier studies showing that Pbx protein localization is regulated during development [69,70]. We were able to make use of pan-cytokeratin expression, a well-established marker of the developing epicardium in many species [39,71,72], to ask whether Pbx proteins are expressed in developing zebrafish proepicardial cells. We noticed that there is a subpopulation of pan-cytokeratin-expressing cells near the ventricle at 48 hpf that is devoid of nuclear Pbx expression (Figure 5). We also noticed a loss of *tbx18* expression in the developing proepicardial region in *pbx4^b557^−/−* embryos at 48 hpf (Figure 4). The proepicardium gives rise to the epicardium, a mesothelial lining of the heart that is needed for proper heart and coronary vessel development and that has been implicated in cardiac regeneration [73–75]. The heterogeneity of Pbx expression among pan-cytokeratin expressing cells near the ventricle thus offers a platform for investigating potential actions of Pbx in proepicardial and epicardial cells during cardiac development and regeneration. Whether reduced *tbx18* expression (Figure 4) represents the loss of an epicardial subset, or indicates *tbx18* transcription is directly regulated by Pbx proteins, requires further work. What roles Pbx expression heterogeneity among cytokeratin-positive cells may play in the development and diversity of proepicardial cell fates also remains to be elucidated in future studies.

## 5. Conclusions

We show that zebrafish *pbx4* mutant embryos exhibit delayed onset of myocardial differentiation, delayed myocardial morphogenesis, and dysmorphic patterning of the ventricle and atrium, consistent with our previous Pbx knock-down studies. In addition, we find that *pbx4* mutant larvae have aberrant outflow tracts and defective expression of the proepicardial marker *tbx18*. We also present evidence for Pbx expression in cardiomyocyte precursors as well as heterogeneous Pbx expression among the pan-cytokeratin-expressing proepicardial cells near the developing ventricle. In conclusion, our data show that Pbx4 is required for the proper temporal activation of myocardial differentiation and establish a basis for studying additional roles of Pbx factors in heart development.

## References

[1] Yelon D (2001). Cardiac patterning and morphogenesis in zebrafish. Dev. Dyn.

[2] Evans SM, Yelon D, Conlon FL, Kirby ML (2010). Myocardial lineage development. Circ. Res.

[3] Kupperman E, Songzhu A, Osborne N, Waldron S, Stainier DYR (2000). A sphingosine-1-phosphate receptor regulates cell migration during vertebrate heart development. Nature.

[4] Glickman NS, Yelon D (2002). Cardiac development in zebrafish: Coordination of form and function. Semin. Cell Dev. Biol.

[5] Bakkers J, Verhoeven MC, Abdelilah-Seyfried S (2009). Shaping the zebrafish heart: From left-right axis specification to epithelial tissue morphogenesis. Dev. Biol.

[6] Stankunas K, Shang C, Twu KY, Kao SC, Jenkins NA, Copeland NG, Sanyal M, Selleri L, Cleary ML, Chang CP (2008). Pbx/Meis deficiencies demonstrate multigenetic origins of congenital heart disease. Circ. Res.

[7] Chang CP, Stankunas K, Shang C, Kao SC, Twu KY, Cleary ML (2008). Pbx1 functions in distinct regulatory networks to pattern the great arteries and cardiac outflow tract. Development.

[8] Maves L, Tyler A, Moens CB, Tapscott SJ (2009). Pbx acts with Hand2 in early myocardial differentiation. Dev. Biol.

[9] Paige SL, Thomas S, Stoick-Cooper CL, Wang H, Maves L, Sandstrom R, Pabon L, Reinecke H, Pratt G, Keller G (2012). A temporal chromatin signature in human embryonic stem cells identifies regulators of cardiac development. Cell.

[10] Wamstad JA, Alexander JM, Truty RM, Shrikumar A, Li F, Eilertson KE, Ding H, Wylie JN, Pico AR, Capra JA (2012). Dynamic and coordinated epigenetic regulation of developmental transitions in the cardiac lineage. Cell.

[11] Mahmoud AI, Kocabas F, Muralidhar SA, Kimura W, Koura AS, Thet S, Porrello ER, Sadek HA (2013). Meis1 regulates postnatal cardiomyocyte cell cycle arrest. Nature.

[12] Waskiewicz AJ, Rikhof HA, Moens CB (2002). Eliminating zebrafish pbx proteins reveals a hindbrain ground state. Dev. Cell.

[13] Moens CB, Selleri L (2006). Hox cofactors in vertebrate development. Dev. Biol.

[14] Berkes CA, Bergstrom DA, Penn BH, Seaver KJ, Knoepfler PS, Tapscott SJ (2004). Pbx marks genes for activation by MyoD indicating a role for a homeodomain protein in establishing myogenic potential. Mol. Cell.

[15] Maves L, Waskiewicz AJ, Paul B, Cao Y, Tyler A, Moens CB, Tapscott SJ (2007). Pbx homeodomain proteins direct Myod activity to promote fast-muscle differentiation. Development.

[16] Fong AP, Yao Z, Zhong JW, Johnson NM, Farr GH, Maves L, Tapscott SJ (2015). Conversion of MyoD to a neurogenic factor: Binding site specificity determines lineage. Cell Rep.

[17] He A, Kong SW, Ma Q, Pu WT (2011). Co-occupancy by multiple cardiac transcription factors identifies transcriptional enhancers active in heart. Proc. Natl. Acad. Sci. USA.

[18] Westerfield M (2007). The Zebrafish Book. A Guide for the Laboratory Use of Zebrafish (Danio rerio).

[19] Pöpperl H, Rikhof H, Chang H, Haffter P, Kimmel CB, Moens CB (2000). *lazarus* is a novel *pbx* gene that globally mediates *hox* gene function in zebrafish. Mol. Cell.

[20] Huang CJ, Tu CT, Hsiao CD, Hsieh FJ, Tsai HJ (2003). Germ-line transmission of a myocardium-specific GFP transgene reveals critical regulatory elements in the cardiac myosin light chain 2 promoter of zebrafish. Dev. Dyn.

[21] Schumacher JA, Bloomekatz J, Garavito-Aguilar ZV, Yelon D (2013). Tal1 regulates the formation of intercellular junctions and the maintenance of identity in the endocardium. Dev. Biol.

[22] Zhang R, Han P, Yang H, Ouyang K, Lee D, Lin YF, Ocorr K, Kang G, Chen J, Stainier DY (2013). *In vivo* cardiac reprogramming contributes to zebrafish heart regeneration. Nature.

[23] Oxtoby E, Jowett T (1993). Cloning of the zebrafish krox-20 gene (krx-20) and its expression during hindbrain development. Nucleic Acids Res.

[24] Sehnert AJ, Huq A, Weinstein BM, Walker C, Fishman M, Stainier DY (2002). Cardiac troponin T is essential in sarcomere assembly and cardiac contractility. Nat. Genet.

[25] Yelon D, Horne SA, Stainier DY (1999). Restricted expression of cardiac myosin genes reveals regulated aspects of heart tube assembly in zebrafish. Dev. Biol.

[26] Berdougo E, Coleman H, Lee DH, Stainier DY, Yelon D (2003). Mutation of weak atrium/atrial myosin heavy chain disrupts atrial function and influences ventricular morphogenesis in zebrafish. Development.

[27] Reiter JF, Alexander J, Rodaway A, Yelon D, Patient R, Holder N, Stainier DY (1999). Gata5 is required for the development of the heart and endoderm in zebrafish. Genes Dev.

[28] Yelon D, Ticho B, Halpern ME, Ruvinsky I, Ho RK, Silver LM, Stainier DY (2000). The bHLH transcription factor hand2 plays parallel roles in zebrafish heart and pectoral fin development. Development.

[29] Miao M, Bruce AE, Bhanji T, Davis EC, Keeley FW (2007). Differential expression of two tropoelastin genes in zebrafish. Matrix Biol.

[30] Zhou Y, Cashman TJ, Nevis KR, Obregon P, Carney SA, Liu Y, Gu A, Mosimann C, Sondalle S, Peterson RE (2011). Latent TGF-β binding protein 3 identifies a second heart field in zebrafish. Nature.

[31] Talbot JC, Johnson SL, Kimmel CB (2010). Hand2 and Dlx genes specify dorsal, intermediate and ventral domains within zebrafish pharyngeal arches. Development.

[32] Choe SK, Lu P, Nakamura M, Lee J, Sagerström CG (2009). Meis cofactors control HDAC and CBP accessibility at Hox-regulated promoters during zebrafish embryogenesis. Dev. Cell.

[33] Feng X, Adiarte EG, Devoto SH (2006). Hedgehog acts directly on the zebrafish dermomyotome to promote myogenic differentiation. Dev. Biol.

[34] Dent JA, Polson AG, Klymkowsky MW (1989). A whole-mount immunocytochemical analysis of the expression of the intermediate filament protein vimentin in *Xenopus*. Development.

[35] Drummond IA, Davidson AJ (2010). Zebrafish kidney development. Methods Cell Biol.

[36] Fiji http://fiji.sc/Fiji.

[37] Preibisch S, Saalfeld S, Tomancak P (2009). Globally optimal stitching of tiled 3D microscopic image acquisitions. Bioinformatics.

[38] Weinberg ES, Allende ML, Kelly CS, Abdelhamid A, Murakami T, Andermann P, Doerre OG, Grunwald DJ, Riggleman B (1996). Developmental regulation of zebrafish MyoD in wild-type, no tail and spadetail embryos. Development.

[39] Kraus F, Haenig B, Kispert A (2001). Cloning and expression analysis of the mouse T-box gene Tbx18. Mech. Dev.

[40] Liu J, Stainier DY (2010). Tbx5 and Bmp signaling are essential for proepicardium specification in zebrafish. Circ. Res.

[41] Hami D, Grimes AC, Tsai HJ, Kirby ML (2011). Zebrafish cardiac development requires a conserved secondary heart field. Development.

[42] Lazic S, Scott IC (2011). Mef2cb regulates late myocardial cell addition from a second heart field-like population of progenitors in zebrafish. Dev. Biol.

[43] Hinits Y, Pan L, Walker C, Dowd J, Moens CB, Hughes SM (2012). Zebrafish Mef2ca and Mef2cb are essential for both first and second heart field cardiomyocyte differentiation. Dev. Biol.

[44] Guner-Ataman B, Paffett-Lugassy N, Adams MS, Nevis KR, Jahangiri L, Obregon P, Kikuchi K, Poss KD, Burns CE, Burns CG (2013). Zebrafish second heart field development relies on progenitor specification in anterior lateral plate mesoderm and nkx2.5 function. Development.

[45] Nevis K, Obregon P, Walsh C, Guner-Ataman B, Burns CG, Burns CE (2013). Tbx1 is required for second heart field proliferation in zebrafish. Dev. Dyn.

[46] Zeng XX, Yelon D (2014). Cadm4 restricts the production of cardiac outflow tract progenitor cells. Cell Rep.

[47] Peralta M, Steed E, Harlepp S, González-Rosa JM, Monduc F, Ariza-Cosano A, Cortés A, Rayón T, Gómez-Skarmeta JL, Zapata A (2013). Heartbeat-driven pericardiac fluid forces contribute to epicardium morphogenesis. Curr. Biol.

[48] Trinh LA, Yelon D, Stainier DY (2005). Hand2 regulates epithelial formation during myocardial differentiation. Curr. Biol.

[49] Garavito-Aguilar ZV, Riley HE, Yelon D (2010). Hand2 ensures an appropriate environment for cardiac fusion by limiting Fibronectin function. Development.

[50] Cerdá-Esteban N, Spagnoli FM (2014). Glimpse into Hox and tale regulation of cell differentiation and reprogramming. Dev. Dyn.

[51] De la Serna IL, Ohkawa Y, Berkes CA, Bergstrom DA, Dacwag CS, Tapscott SJ, Imbalzano AN (2005). MyoD targets chromatin remodeling complexes to the myogenin locus prior to forming a stable DNA-bound complex. Mol. Cell. Biol.

[52] Bryantsev AL, Duong S, Brunetti TM, Chechenova MB, Lovato TL, Nelson C, Shaw E, Uhl JD, Gebelein B, Cripps RM (2012). Extradenticle and homothorax control adult muscle fiber identity in Drosophila. Dev. Cell.

[53] Yao Z, Farr GH, Tapscott SJ, Maves L (2013). Pbx and Prdm1a transcription factors differentially regulate subsets of the fast skeletal muscle program in zebrafish. Biol. Open.

[54] Cho OH, Mallappa C, Hernández-Hernández JM, Rivera-Pérez JA, Imbalzano AN (2015). Contrasting roles for MyoD in organizing myogenic promoter structures during embryonic skeletal muscle development. Dev. Dyn.

[55] Capellini TD, di Giacomo G, Salsi V, Brendolan A, Ferretti E, Srivastava D, Zappavigna V, Selleri L (2006). Pbx1/Pbx2 requirement for distal limb patterning is mediated by the hierarchical control of Hox gene spatial distribution and Shh expression. Development.

[56] Ferretti E, Li B, Zewdu R, Wells V, Hebert JM, Karner C, Anderson MJ, Williams T, Dixon J, Dixon MJ (2011). A conserved Pbx-Wnt-p63-Irf6 regulatory module controls face morphogenesis by promoting epithelial apoptosis. Dev. Cell.

[57] Koss M, Bolze A, Brendolan A, Saggese M, Capellini TD, Bojilova E, Boisson B, Prall OW, Elliott DA, Solloway M (2012). Congenital asplenia in mice and humans with mutations in a Pbx/Nkx2-5/p15 module. Dev. Cell.

[58] Farr GH, Maves L (2015). Seattle Children’s Research Institute, Seattle, WA, USA. Unpublished work.

[59] Kok FO, Shin M, Ni CW, Gupta A, Grosse AS, van Impel A, Kirchmaier BC, Peterson-Maduro J, Kourkoulis G, Male I (2015). Reverse genetic screening reveals poor correlation between morpholino-induced and mutant phenotypes in zebrafish. Dev. Cell.

[60] Rossi A, Kontarakis Z, Gerri C, Nolte H, Hölper S, Krüger M, Stainier DY (2015). Genetic compensation induced by deleterious mutations but not gene knockdowns. Nature.

[61] Hutson MR, Kirby ML (2003). Neural crest and cardiovascular development: A 20-year perspective. Birth Defects Res. C Embryo Today.

[62] Prall OW, Menon MK, Solloway MJ, Watanabe Y, Zaffran S, Bajolle F, Biben C, McBride JJ, Robertson BR, Chaulet H (2007). An Nkx2-5/Bmp2/Smad1 negative feedback loop controls heart progenitor specification and proliferation. Cell.

[63] De Pater E, Ciampricotti M, Priller F, Veerkamp J, Strate I, Smith K, Lagendijk AK, Schilling TF, Herzog W, Abdelilah-Seyfried S (2012). Bmp signaling exerts opposite effects on cardiac differentiation. Circ Res.

[64] Hurtado R, Zewdu R, Mtui J, Liang C, Aho R, Kurylo C, Selleri L, Herzlinger D (2015). Pbx1-dependent control of VMC differentiation kinetics underlies gross renal vascular patterning. Development.

[65] Lu J, Landerholm TE, Wei JS, Dong XR, Wu SP, Liu X, Nagata K, Inagaki M, Majesky MW (2001). Coronary smooth muscle differentiation from proepicardial cells requires rhoA-mediated actin reorganization and p160 rho-kinase activity. Dev. Biol.

[66] Richarte AM, Mead HB, Tallquist MD (2007). Cooperation between the PDGF receptors in cardiac neural crest cell migration. Dev. Biol.

[67] Mellgren AM, Smith CL, Olsen GS, Eskiocak B, Zhou B, Kazi MN, Ruiz FR, Pu WT, Tallquist MD (2008). Platelet-derived growth factor receptor beta signaling is required for efficient epicardial cell migration and development of two distinct coronary vascular smooth muscle cell populations. Circ. Res.

[68] Arrington CB, Dowse BR, Bleyl SB, Bowles NE (2012). Non-synonymous variants in pre-B cell leukemia homeobox (PBX) genes are associated with congenital heart defects. Eur. J. Med. Genet.

[69] Mann RS, Abu-Shaar M (1996). Nuclear import of the homeodomain protein extradenticle in response to Wg and Dpp signaling. Nature.

[70] Gonzálaz-Crespo S, Abu-Shaar M, Torres M, Martínez-A C, Mann RS, Morata G (1998). Antagonism between extradenticle function and Hedgehog signaling in the developing limb. Nature.

[71] Vrancken Peeters MP, Mentink MM, Poelmann RE, Gittenberger-de Groot AC (1995). Cytokeratins as a marker for epicardial formation in the quail embryo. Anat. Embryol.

[72] Dettman RW, Denetclaw W, Ordahl CP, Bristow J (1998). Common epicardial origin of coronary vascular smooth muscle, perivascular fibroblasts, and intermyocardial fibroblasts in the avian heart. Dev. Biol.

[73] Schlueter J, Brand T (2012). Epicardial progenitor cells in cardiac development and regeneration. J. Cardiovasc. Transl. Res.

[74] Wu SP, Dong XR, Regan JN, Su C, Majesky MW (2013). *Tbx18* regulates development of the epicardium and coronary vessels. Dev. Biol.

[75] Masters M, Riley PR (2014). The epicardium signals the way towards heart regeneration. Stem Cell Res.

